# A case of protein S-specific activity triggers detection with potential thrombosis development

**DOI:** 10.1016/j.jvscit.2025.102105

**Published:** 2025-12-17

**Authors:** Hideaki Yamada, Mitsumasa Ohgi, Naoki Tominaga, Akira Tsujimoto, Akiyoshi Fujishima, Shinya Matsumoto

**Affiliations:** a Department of Vascular Surgery, Shinkomonji Hospital, Kitakyushu, Japan; b Department of Clinical Laboratory, Shinkomonji Hospital, Kitakyushu, Japan; c Department of Internal Medicine, Shinkomonji Hospital, Kitakyushu, Japan; d Department of Pharmaceutics, Shinkomonji Hospital, Kitakyushu, Japan; e Department of Clinical Chemistry and Laboratory Medicine, Kyushu University Hospital, Fukuoka, Japan

**Keywords:** Deep vein thrombosis, Protein S, Protein S Tokushima

## Abstract

Protein S gene abnormalities are the most common congenital predisposition to thrombophilia in the Japanese population, but not in the Caucasian population. It is important to measure protein S activity, specific activity, and antigen levels in patients with thrombophilia. A 52-year-old man presented with suspected deep vein thrombosis after previously visiting an orthopedic clinic with right lower extremity swelling after his long-distance walk 5 days prior. His D-dimer level was elevated, and a thrombus was found from the femoral to the below-the-knee veins on ultrasonographic echography. Anticoagulation therapy was initiated. His parents had previously been diagnosed with deep vein thrombosis; therefore, he was examined for thrombophilic predisposition. The results showed normal protein S activity and antigen levels, but decreased protein S-specific activity. Genetic testing revealed the presence of a protein S variant (protein S Tokushima). In addition to measuring protein activity and antigen levels, protein S-specific activity measurements can reveal increased risks of thrombosis.

Venous thromboembolism is the most frequently encountered form of thrombosis in clinical practice because of its genetic and acquired risk factors. Venous thromboembolism is a potentially life-threatening condition,^1^^,^^2^ and the protein S gene variant is reported as the most common congenital thrombophilic predisposition in the Japanese population, whereas it is relatively uncommon in the Caucasian population.^3^ Given these regional differences, it is particularly important to screen patients with a family history of thrombosis or those who develop thrombosis without any risk factors. The genetic mutation associated with protein S deficiency, characterized by reduced activity at normal protein levels, is most common in the Japanese population.^4^ Therefore, it is common practice to examine total protein S antigen and activity. Furthermore, the importance of measuring protein S specific activity (ie, total protein S activity/total protein S antigen ratio) has been demonstrated in recent years. Protein S-specific activity is important to monitor hypercoagulable states more appropriately because its physiological and pathological effects can alter protein S activity and antigen levels.^5^^,^^6^

We recently encountered a case of deep vein thrombosis (DVT), highlighting reduced protein S-specific activity despite normal protein S and antigen levels as a potential pathology in patients with protein S Tokushima variant.

## Patient presentation

A 52-year-old man was referred to our department with suspected DVT after presenting to an orthopedic clinic with right lower extremity swelling that developed after walking a long distance 5 days prior. His D-dimer level was elevated, and a thrombus was found from the femoral vein to the below-the-knee veins on ultrasonographic echography. The patient was initiated on anticoagulation therapy with edoxaban, a direct oral anticoagulant. His parents had a history of DVT; therefore, he was examined for thrombophilic predisposition. The results showed that protein S activity and antigen levels were normal; however, a reduction in protein S-specific activity was observed. Genetic testing revealed an abnormality in the patient's S protein gene. He had previously undergone colorectal cancer surgery 18 months earlier for early-stage disease, and only periodic follow-up was required. The patient had no history of thrombosis. He had a smoking history of 20 cigarettes per day for 21 years and drank occasionally.

The patient's vital signs were stable. His body mass index was 21.6 kg/m^2^, and his body surface area was 1.92 m^2^. Jugular veins were not distended. Cardiac examinations revealed no murmurs or gallops. The right lower extremity was swollen, and tenderness was noted in the sural region. The patient was positive for the Homan's sign. Abnormal basic laboratory test findings included elevated fibrin degradation product (74.5 μg/mL; reference, <5.0 μg/mL) and D-dimer (13.7 μg/mL; reference, <0.5 μg/mL) levels (Table). Other blood test results, including white blood cells, hemoglobin, platelets, creatinine, albumin, prothrombin time, and activated partial thromboplastin time, were all within normal ranges. The patient revealed that his parents had experienced DVT. Therefore, the patient was tested for thrombogenic predisposition, which revealed normal protein S, antigen, and protein C, antithrombin, lupus anticoagulant, and antiphospholipid antibody levels. Notably, only the protein S-specific activity (activity/antigen ratio) level was decreased to 0.71 (1.12/1.57) IU/mL (reference, <0.86-1.18 IU/mL) (Table). Therefore, the patient was diagnosed with a qualitative protein S abnormality (type II with reduced protein S activity only). Subsequent genetic testing identified an abnormality in the patient's protein S gene, which comprised a single-base substitution in exon 6 that caused a missense mutation called the protein S Tokushima variant, the most common gene variant. These mutations were heterozygous. As scientific evidence of heredity typically requires demonstration across at least three generations of similar genetic abnormalities, we recommend genetic testing of the patient's parents and children.

**Table:** Blood test findings at the onset of deep vein thrombosis (DVT)

Coagulant and thrombophilia testing at onset		
Variable	Value	Reference interval
Bleeding time	1.5 min	<5 min
PT%	108%	70%-130%
PT-INR	0.96	0.85-1.15
APTT	26.0 s	26.0-38.0 s
Fibrinogen	268 mg/dL	200-400 mg/dL
FDP	74.5 μg/mL	<10.0 μg/mL
D-dimer	13.7 μg/mL	<1.0 μg/mL
Lupus anticoagulant	0.95	<1.16
Anticardiolipin IgG antibody	4 U/mL	<12.3 U/mL
Protein C activity	158%	70%-140%
Antithrombin activity	122%	80%-130%
Protein S antigen	1.57 IU/mL	0.80-1.31 IU/mL
Protein S activity	1.12 IU/mL	0.82-1.38 IU/mL
Protein S-specific activity	0.71 IU/mL	0.86-1.18 IU/mL

*APTT,* Activated partial thromboplastin time; *FDP,* fibrin degradation products; *INR,* international normalized ratio; *PT,* prothrombin time.

## Discussion

We recently encountered a case of DVT in which protein S activity and antigen levels were normal; however, the protein S-specific activity, when calculated, showed a marked decrease (Table). This implies that a relative decrease exists in protein S activity. The patient was subsequently diagnosed with a protein S Tokushima gene variant.

Protein S, together with protein C, promotes anticoagulant activity as a cofactor for activated protein C. Protein S abnormalities have the highest prevalence among Japanese individuals, affecting 1% to 3% of the population. Protein S abnormalities are less common (0.03%-0.13%) in the Caucasian population.^7^ On the other hand, the Factor V Leiden variant, which causes the activated protein C resistance, is a major genetic risk factor in the Caucasian population, whereas it is not present in the Japanese population.^8^^,^^9^ Protein S Tokushima (p.Lys196Glu, rs121918474), which has an allele frequency of 0.9% in the Japanese population, is the most common and highly prevalent gene variant with a qualitative (type II with reduced protein S activity only) defective plasma phenotype. The genetic risk factors for DVT include protein S Tokushima (p.Lys196Glu, rs121918474), and it has been shown to increase the risk of DVT by 3.7 to 8.6 times compared with healthy controls.^3^^,^^10^^,^^11^

The diagnosis of protein S abnormalities begins with blood tests to measure the antigen levels (total and free forms) and activity of protein S. If these measurements fall below the reference range, genetic testing is performed to identify mutations in the protein S gene, thereby confirming the diagnosis. However, both protein S antigen and activity are affected by physiological (eg, sex, age, and hormonal changes such as those occurring during menstruation) and pathological (eg, infection) factors.^12^ In recent years, it has been demonstrated that measuring protein S-specific activity is crucial for addressing the issue.^5^^,^^6^ The specific activity levels of protein S (ie, total protein S activity/total protein S antigen ratio) should be measured to monitor hypercoagulable states more appropriately and screen for abnormalities in the protein S gene.^4^, ^5^, ^6^ If the protein S-specific activity was <0.78, there was a high possibility of a genetic abnormality.^5^^,^^6^ In this case, the protein S-specific activity was <0.78, and as a result of genetic testing, the patient was diagnosed with a genetic variant of protein S Tokushima. In addition to the protein S Tokushima mutation, the patient harbored thrombotic risk factors due to his history of cancer and smoking. The patient's history of cancer within the last 5 years and his history of smoking may have contributed to the development of thrombosis.

## Conclusions

Blood samples with decreased protein S-specific activity may increase the risk of developing thrombosis, even if the activity and antigen levels are normal. Therefore, it is important to measure the specific activity as well, as protein S abnormalities may be missed by testing protein S activity and antigen levels alone.

## Funding

None.

## Patient consent

Informed consent was obtained from the patients for participation in this case report and for the publication of their medical information. This case report was approved by the appropriate institutional review board, and the investigation was carried out in accordance with the Declaration of Helsinki.

## Disclosures

None.

## References

[1] Rosendaal F.R. (2005). Venous thrombosis: the role of genes, environment, and behavior. Hematol Am Soc Hematol Educ Program.

[2] Zakai N.A., McClure L.A. (2011). Racial differences in venous thromboembolism. J Thromb Haemost.

[3] Kinoshita S., Iida H., Inoue S. (2005). Protein S and protein C gene mutations in Japanese deep vein thrombosis patients. Clin Biochem.

[4] Hamasaki N., Kuma H., Tsuda H. (2013). Activated protein C anticoagulant system dysfunction and thrombophilia in Asia. Ann Lab Med.

[5] Jin X., Kinoshita S., Kuma H. (2021). Reduced activity of protein S in plasma: a risk factor for venous thromboembolism in the Japanese population. Clin Appl Thromb Hemost.

[6] Dai Ohira, Seiichiro Kamimura, Seiichiro Kamimura, Naotaka Hamasaki (2025). Contrasting etiology of thrombophilia in Asians and Caucasians. Ann Clin Case Rep.

[7] Roberts L.N., Patel R.K., Arya R. (2009). Venous thromboembolism and ethnicity. Br J Haematol.

[8] Nakamura M, Yamada N, Ito M (2015). Current management of venous thromboembolism in Japan: current epidemiology and advances in anticoagulant therapy. J Cardiol.

[9] Ro A., Hara M., Takada A. (1999). The factor V Leiden mutation and the prothrombin G20210A mutation was not found in Japanese patients with pulmonary thromboembolism. Thromb Haemost.

[10] Kimura T., Honda S., Kawasaki T. (2006). S-K196E mutation as a genetic risk factor for deep vein thrombosis in Japanese patients. Blood.

[11] Ikejiri M., Wada H., Sakamoto Y. (2010). The association of protein S Tokushima-K196E with a risk of deep vein thrombosis. Int J Hematol.

[12] Noguchi K., Nakazono E., Tsuda T. (2019). Plasma phenotypes of protein S Lys196Glu and protein C Lys193del variants prevalent among young Japanese women. Blood Coagul Fibrinolysis.

